# The evolutionary origin of the need to sleep: an inevitable consequence of synaptic neurotransmission?

**DOI:** 10.3389/fnsyn.2015.00015

**Published:** 2015-09-22

**Authors:** Robert S. Cantor

**Affiliations:** ^1^Burke Laboratory, Department of Chemistry, Dartmouth CollegeHanover, NH, USA; ^2^Memphys Center for Biomembrane Physics, University of Southern DenmarkOdense, Denmark

**Keywords:** neurotransmitter, postsynaptic receptor, synapse, bilayer, ISF, glymphatic system, sleep

## Abstract

It is proposed that the evolutionary origin of the need to sleep is the removal of neurotransmitters (NTs) that escape reuptake and accumulate in brain interstitial fluid (ISF). Recent work suggests that the activity of ionotropic postsynaptic receptors, rapidly initiated by binding of NTs to extracellular sites, is modulated over longer times by adsorption of these NTs to the lipid bilayers in which the receptors are embedded. This bilayer-mediated mechanism is far less molecularly specific than binding, so bilayer adsorption of NTs that have diffused into synapses for other receptors would modulate their activity as well. Although NTs are recycled by membrane protein reuptake, the process is less than 100% efficient; a fraction escapes the region in which these specific reuptake proteins are localized and eventually diffuses throughout the ISF. It is estimated that even if only 0.1% of NTs escape reuptake, they would accumulate and adsorb to bilayers in synapses of other receptors sufficiently to affect receptor activity, the harmful consequences of which are avoided by sleep: a period of efficient convective clearance of solutes together with greatly reduced synaptic activity.

In fast synaptic transmission, the exocytotic fusion of a vesicle with the presynaptic membrane releases thousands of neurotransmitter (NT) molecules that diffuse into the synaptic cleft, a small fraction of which encounter and bind to specific sites on their receptors in the postsynaptic membrane. Reuptake mechanisms involving membrane transport proteins (as well as enzymatic degradation of some NTs) eliminate a large fraction of these NTs either within or near the synapse, preventing their local accumulation. The efficacy of this elimination/recycling process—which relies on diffusional encounters of NTs with membrane proteins specific to that NT—is less than 100%: some small fraction of the NTs escape by diffusing out of the region. Although the concentration of a particular NT would remain very low at the synapses and nearby glia where its selective reuptake proteins are localized and functioning, its concentration would be expected gradually to increase elsewhere throughout the entire interstitial fluid (ISF) network, if clearance (through diffusive or convective flow) of solutes out of the ISF is sufficiently slow. Simply stated, in the vicinity of a synapse that is specific to a single NT, the concentration of all of the *other* NTs would be expected gradually to rise.

How might this increased concentration of NTs affect synaptic activity? The extracellular activation sites on a postsynaptic receptor are specific to one NT, so if binding to those sites were the only manner in which NTs could influence receptor activity, the receptor would be unaffected by the presence of other “noncognate” NTs. However, it has been proposed (Cantor, 2003) that NTs modulate the activity of their receptors not only by binding to these extracellular sites but also indirectly, by adsorbing to the lipid bilayers of the postsynaptic membranes in which the receptors are embedded. This indirect mechanism differs from direct binding in that it has far less molecular specificity; adsorption of a very wide range of solutes, including all the NTs, as well as other interfacially active compounds such as anesthetics, would be expected to modulate the activity of postsynaptic receptors. So, if the concentration of NTs throughout the ISF gradually rises, their adsorption onto postsynaptic bilayers will increase as well, to a degree determined by their adsorption equilibrium constants. The lack of molecular specificity means that the net effect on any one postsynaptic receptor arises from contributions from *all* the noncognate NTs (as well as elevated levels of metabolites, etc.)

What underlies this bilayer-mediated mechanism, and what evidence exists to support it? Adsorption of solutes alters the physical properties of the bilayer, which distorts the conformational free energy landscape of the receptor, and thus alters the equilibrium and rate constants of its conformational transitions (Cantor et al., 2009; Sonner and Cantor, 2013; Lee et al., 2015). Bilayer adsorption/desorption of NTs—with kinetics independent of the kinetics of binding/unbinding—thus shapes the time-dependence of the open-channel probability after rapid initial activation by binding. Since this adaptation allows for sensitive tuning of desensitization, a critical determinant of the total ion flux, it presumably confers a significant selective advantage to the organism. As a challenging test of this hypothesis, it has been shown (Cantor et al., 2009; Lee et al., 2015) that all of the complex features of current traces observed in electrophysiological studies on recombinant GABA_A_ receptors can be reproduced by a simple kinetic scheme with only a minimal set of protein conformational states, but which incorporates bilayer adsorption; indeed, it was demonstrated that the full range of features cannot be reproduced with a classical direct binding approach unless multiple additional hypothetical (and unmeasurable) conformational states are incorporated into the scheme. Finally, more generally, bilayer incorporation of solutes (and changes in lipid composition) has long been known to affect membrane proteins, such as rhodopsin (Mitchell et al., 1996), whose activity depends on conformational transitions, but which is not initiated by ligand binding.

The clearance of solutes through the cerebrospinal fluid (CSF) and ISF during awake periods has been shown to be extremely slow, so in light of the above discussion, some other mechanism of clearance of NTs from the ISF would be necessary to maintain proper function of the central nervous system (CNS; Iliff et al., 2012). One possibility would be regular periods in which the rate of clearance of solutes from the ISF is greatly increased, during which synaptic transmission is significantly reduced, of sufficient duration to ensure the elimination of NTs from the ISF. Sleep would provide this function, if accompanied by a large increase in the rate of solute clearance; if so, it suggests the evolutionary origin of this “need to sleep”. The plausibility of this suggestion is strongly supported by recent studies (Xie et al., 2013) that have demonstrated that sleep is indeed characterized by greatly enhanced convective flow through the CSF and ISF, presumably clearing not only NTs but also many potentially harmful products of neuronal metabolism from the ISF.

In our recent study on GABA_A_ receptors (Lee et al., 2015), it was shown that bilayer-mediated effects of GABA—as predicted from the kinetic model, in agreement with a broad range of electrophysiological results—become significant as the aqueous concentration of GABA approaches 1 mM. If other NTs have similar partition coefficients, then in order for NT adsorption to have a substantial effect on the activity of any postsynaptic receptor, the total NT concentration would need to approach a similar value, i.e., of order 1 mM. In light of the above discussion, the question arises: for a typical awake period, how inefficient would the reuptake mechanisms have to be (i.e., the maximum fraction of NTs that escape their cognate synaptic regions) before bilayer-adsorbed NTs start to alter the activity of postsynaptic receptors?

A crude, order-of-magnitude estimate of this efficiency is obtained by assuming that NTs that are not consumed by reuptake or enzymatic degradation diffuse to a roughly uniform concentration (except near synapses for their own receptors), and that while awake, they are not cleared from the ISF through diffusive or convective flow. It depends on a set of parameters, estimated below, that characterize the adult brain. If *F* represents the fraction of NTs that survive in the ISF, i.e., that are *not* eliminated by reuptake, enzymatic degradation, or diffusion out of ISF, then

$$F\approx cN_{\text{Av }}V_{\text{ISF}}/(n_{\text{NT }}N_{\text{fs }}n_{\text{ves }}vt)$$

where *t* ≈ 16 h is the duration of an awake period, *c* is the average extracellular concentration of NT molecules at the end of that period, *V*_ISF_ is the volume of ISF, *n*_NT_ is the number of NT molecules emitted during vesicle exocytosis, *N*_fs_ is an estimate of the number of fast synapses in the brain, *v* represents the average frequency at which action potentials arrive at a presynaptic membrane, *n*_ves_ is the average number of vesicles that undergo exocytosis in response to the arrival of an action potential, and *N*_Av_ is Avogadro’s number. The values of these parameters can be estimated, some fairly precisely but others only to within an order of magnitude. Xie et al. (2013) report that during awake periods, the ISF comprises 14% of murine brain volume, consistent with earlier estimates (Doczi, 1993). With typical human adult brain volume ≈ 1.1 L, *V*_ISF_ ≈ 0.15L. Estimates in the literature of the number of NTs per vesicle range from 2500 to 5000 for glutamate (Riveros et al., 1986) and GABA (Telgkamp et al., 2004), 4700 for serotonin (Bruns and Jahn, 1995), and higher for acetylcholine, so *n*_NT_ ≈ 4000 is chosen as a representative value. The number of synapses in the human brain is generally estimated at 10^14^–10^15^, and since a significant fraction of these are fast, *N*_syn_ ~ 10^14^ is a reasonable estimate. The frequency of arrival of action potentials at the synapse varies widely (depending both on the type of neuron and variation in neural activity) ranging from *v* ≈ 50 s^−1^ as a typical basal rate for some GABAergic synapses (Gilbert and Thach, 1977; Telgkamp et al., 2004) down to nearly an order of magnitude less elsewhere; *v* ≈ 15 s^−1^ is taken as a representative value. Finally, although the probability of vesicle release is less than 100%, it is counterbalanced by possible release at multiple sites, so *n*_ves_ ≈ 1. Substituting *c* ≈ 1 mM and these estimates of parameter values into the above equation yields *F* ≈ 3 × 10^–4^, i.e., the total concentration of NTs is estimated to reach 1 mM by the end of an awake period if only ~0.03% of the NTs released into synapses through exocytosis escape elimination through reuptake or enzymatic degradation. This estimate assumes negligible clearance of NTs out of the ISF during a wakeful period by either diffusive or convective flow. However, even were some clearance to occur during awake periods, *F* would still be very small, e.g., if 90% of the NTs that survive reuptake were cleared from the ISF, *F* would still be less than 10^–2^. Although only an order-of-magnitude estimate, it nonetheless argues strongly that regular clearance of NTs from the ISF is of paramount importance, which suggests that this need may be the evolutionary origin of sleep: a periodic, prolonged state in which an enormous increase in the rate of clearance of solutes (through convective flow) is coupled with reduced fast synaptic activity.

The above arguments are predicated on two key assumptions: (a) that fast NTs adsorb to postsynaptic membrane bilayers; and (b) that they thereby modulate the behavior of receptors. What evidence exists in support of these assumptions? With regard to the first, Wang et al. (2011) used thermodynamic measurements to show that various fast NTs do indeed interact with bilayers, although the extent of the interaction depends both on the charge state of the NT and on lipid head group composition; the presence of anionic lipids was found to play an important role for zwitterionic and cationic NTs. An earlier study (Rolandi et al., 1990) reported strong interactions between GABA and bilayers composed of an anionic lipid (phosphatidylserine). Bilayer interactions of other NTs such as serotonin and dopamine have been shown to be unexpectedly strong (Peters et al., 2013; Jodko-Piórecka and Litwinienko, 2013).

Experimental evidence in support of the second assumption (that bilayer-adsorbed NTs modulate the activity of postsynaptic receptors) was provided by electrophysiological studies (Milutinovic et al., 2007), which clearly showed that the presence of other fast NTs strongly affects the response of receptors to their own NTs. As there are no known binding sites for most noncognate NTs anywhere on the receptors, it supports (although it does not require) a bilayer-mediated mechanism by which NTs nonspecifically influence receptor activity. And as described above, additional support comes from the success of a kinetic model (Cantor et al., 2009; Lee et al., 2015) in predicting the remarkably complex features of electrophysiological traces observed in postsynaptic receptors such as GABA_A_R, over a broad range of agonist and anesthetic concentrations. Although it incorporates only three protein conformational states (resting, conducting, and desensitized), the model allows for the modulation of the conformational free energy landscape by bilayer adsorption of aqueous solutes, in simple Langmuir approximation. It is capable of reproducing the temporally complex desensitization and deactivation in response to a pulse of agonist, the modulation of those features by volatile anesthetics over a wide range of concentrations (both coapplied with agonist and continuously present), and the activation of receptors by supraclinical anesthetic concentrations in the absence of agonist. In those studies, parameters were determined only for the bilayer-mediated influence of GABA on GABA_A_ receptors, since detailed kinetic data for the effects of noncognate NTs are not available. So, to get a sense for the effect of a different NT on GABA_A_R, calculations have been performed assuming that the noncognate NT and GABA have similar effects on the bilayer (and thus the same values of the relevant kinetic parameters), but the noncognate NT is unable to bind to the receptor’s activation sites. The results are shown in Figure 1: *f_o_(t)*, the fraction of receptors in the open (ion-conducting) conformation as a function of time, is predicted in response to a short (10 ms) pulse of a saturating concentration of GABA on a patch of identical GABA_A_ receptors, for varying concentrations (*c*) of continuously present noncognate NTs. The decrease in the initial peak and the increased rate of deactivation with increasing concentration both contribute to a decrease in the total ion flux *Q*(*c*), i.e., the integrated current.

**Figure 1:**
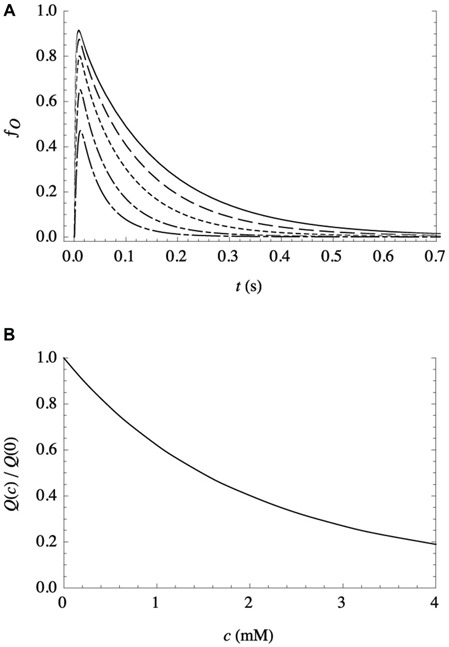
**Effect of a continuously present noncognate NT, at varying concentrations *c*, on the response of a patch of recombinant receptors to a short (10 ms) pulse of a saturating concentration of their NT agonist, as calculated from the kinetic model with parameter values as detailed in Lee et al. (2015). (A)** Representative traces, expressed as the fraction of receptors in the conducting state *vs*. time, for *c* = 0 mM (solid line), 0.5 mM (dashed line), 1.2 mM (dotted line), 2.4 mM (dash-dotted line), and 4.0 mM (long dash-dotted line). **(B)** Total ion flux (i.e., the integrated current) relative to ion flux in the absence of noncognate NT, as a function of *c*.

This reduction in inhibitory (anion) flux is the opposite of the observed and predicted effect of volatile anesthetics, which increase ion flux through inhibitory receptors (Lee et al., 2015). Effects of bilayer adsorption of multiple solutes on the protein free energy landscape are predicted to be additive, which suggests that bilayer incorporation of an anesthetic would offset the effect of a noncognate NT. It is thus tempting to speculate that the reason that oleamide—which has anesthetic-like effects on GABA and glycine receptors (Yost et al., 1998; Coyne et al., 2002) and induces sleep (Huitrón-Reséndiz et al., 2001)—is found at elevated levels in the CSF of sleep-deprived cats (Cravatt et al., 1995) is that its incorporation into bilayers would offset the harmful effects of noncognate NTs.

What experiments could serve to test this hypothesis? The NT composition of ISF could be measured to determine if it is indeed high at the beginning of sleep (and enhanced by sleep deprivation) then gradually decreasing to a low threshold level at waking. Also, infusion of NTs at concentrations well below their binding affinities (or of related compounds that do not bind to *any* of the fast receptors) toward the end of a sleep period would be predicted to affect behavior profoundly during the subsequent awake period. A key biophysical study would determine the equilibrium constants of adsorption of NTs to bilayers with lipid composition similar to their postsynaptic membranes.

Although sleep may be a very efficient adaptation to allow for removal of NTs, it may not be the only possible mechanism by which this clearance can be achieved. For example, in organisms for which predation is an extreme risk over an extended period, a different mechanism might confer a *net* advantage, even if NTs are less efficiently removed, if it significantly reduces predation risk. Thus, whereas regular sleep is necessary under most conditions for the vast majority of species, the existence of exceptions is neither surprising nor provides evidence against this hypothesis. Alternating unihemispherical sleep in marine mammals may be a relevant example, along with the reduction in sleep arising from the need to escape predation during migration, both in birds and in some marine mammals, such as mothers and calves of smaller cetaceans (Kavanau, 1997; Allada and Siegel, 2008; Siegel, 2008). If these organisms have evolved alternative mechanisms to increase ISF clearance during these long periods, even if less efficient than sleep, it could provide a significant net survival advantage.

Various criteria have been used to analyze the strengths and weaknesses of the many hypotheses regarding the origin of the need for sleep (Kavanau, 2005; Allada and Siegel, 2008; Mignot, 2008; Siegel, 2009; Vassalli and Dijk, 2009; Rial et al., 2010; Vyazovskiy and Harris, 2013; Schmidt, 2014; Tononi and Cirelli, 2014). However, there is a significant flaw with regard to the most common of these criteria. The question is often addressed by examining both the beneficial processes that occur during sleep, and the deleterious consequences of sleep deprivation. This approach tacitly presumes that the *origin* of the need to sleep must be related to those processes that are observed to be most essential for survival among the most diverse group of organisms, and to occur uniquely during sleep. This presumption is incorrect. Regardless of the evolutionary origin(s) of the need for sleep, to the degree to which organisms have subsequently evolved to take *additional* advantages of the sleep state, they will have gained a selective survival advantage. Thus, even if some of these additional traits have evolved to become essential for survival—it would be surprising if they had not—they may nonetheless be unrelated to the fundamental need that originally induced the development of sleep early in evolution. A simple example serves to illustrate this point. Consider a collection of financial executives who commute from an exclusive suburban community on an equally exclusive train every morning to their jobs in the financial district of the city. In years past, they did many different things during the morning commute, but with changes in the financial system and the ease of internet access, they could realize a significant selective advantage by using their smartphones or tablets to be informed of late-breaking developments in the markets in advance of their arrival at work. Those who did not, and thus did not enjoy that competitive advantage, eventually lost their jobs and homes in the community, and thus no longer take this train. So, now *every* commuter on the train is engaged with a tablet during the morning trip. Were an anthropologist to perform an experiment in which some subset of the passengers were prevented from working on their tablets over an extended period, those executives would presumably also lose their jobs. Following the conventional logic—based on the behavior observed to occur without exception during the commute, and the harmful consequences of preventing that behavior—the anthropologist would conclude that the origin of the need to take the train is to allow these executives to obtain financial information from their tablets; *a completely erroneous conclusion*, even though this trait has indeed evolved to become essential for their survival. In the context of sleep: its evolutionary origins may be entirely unrelated to most of its present essential functions.

## Conflict of Interest Statement

The author declares that the research was conducted in the absence of any commercial or financial relationships that could be construed as a potential conflict of interest.
